# The age of obesity onset affects changes in subcutaneous adipose tissue macrophages and T cells after weight loss

**DOI:** 10.3389/fimmu.2025.1601847

**Published:** 2025-08-04

**Authors:** Jessica Murphy, José A. Morais, Michael A. Tsoukas, Alexandra B. Cooke, Stella S. Daskalopoulou, Sylvia Santosa

**Affiliations:** ^1^ Department of Health, Kinesiology, and Applied Physiology, Concordia University, Montreal, QC, Canada; ^2^ Metabolism, Obesity, and Nutrition Laboratory, School of Health, Concordia University, Montreal, QC, Canada; ^3^ Centre de recherche - Axe maladies chroniques, Centre intégré universitaire de santé et de services sociaux du Nord-de-l’Ile-de-Montréal, Hôpital du Sacré-Coeur de Montréal, Montreal, QC, Canada; ^4^ Division of Geriatric Medicine, Department of Medicine, McGill University, McGill University Health Centre (MUHC)–Montreal General Hospital, Montreal, QC, Canada; ^5^ Division of Endocrinology, Department of Medicine, McGill University, Royal Victoria Hospital, MUHC Glen site, Montreal, QC, Canada; ^6^ Division of Experimental Medicine, Department of Medicine, McGill University, MUHC Glen site, Montreal, QC, Canada; ^7^ Cardiovascular Health Across the Lifespan (CHAL) Program, Research Institute of the MUHC, Montreal, QC, Canada; ^8^ Division of Internal Medicine, Department of Medicine, McGill University, Royal Victoria Hospital, MUHC Glen site, Montreal, QC, Canada

**Keywords:** obesity, age of onset, subcutaneous adipose tissue, macrophages, T cells, adipokines, inflammation, weight loss

## Abstract

**Introduction:**

Adipose tissue inflammation, driven in part by immune cells, may contribute to the elevated type 2 diabetes risk in adults with childhood-onset obesity (CO) compared to those with adult-onset obesity (AO). Weight loss can modify adipose tissue immune cell composition, but whether these changes differ by obesity onset remains unknown.

**Methods:**

We compared abdominal and femoral subcutaneous adipose tissue (SAT) immune cell percentages between people with CO and AO before and after moderate (~10%) weight loss. We collected abdominal and femoral SAT from females with CO or AO before (CO: *n*=14; AO: *n*=13) and after (CO: *n*=8; AO: *n*=6) diet- and exercise-induced weight loss. We used flow cytometry to quantify the percentages of macrophages and T cells in the stromovascular fraction of both SAT regions.

**Results:**

Abdominal CD68^+^CD206^- ‘^pro-inflammatory’ macrophages were slightly higher in AO than CO at baseline but declined in AO only, equalizing between groups after weight loss. Femoral CD68^+^CD206^-^ macrophages, as well as abdominal and femoral CD68^+^CD206^+^ ‘anti-inflammatory’ macrophages and CD3^+^CD8^+^ T cells, did not differ between groups at baseline or change after weight loss. Abdominal and femoral CD3^+^CD4^+^ T cells—potentially pro- or anti-inflammatory—increased after weight loss in AO but remained unchanged in CO.

**Discussion:**

Our findings, though preliminary, do not support the hypothesis that SAT immune cell profiles account for the elevated type 2 diabetes risk in CO. Weight loss appears to alter some immune cell populations in AO but not in CO. The long-term metabolic consequences of these changes—or lack thereof—remain to be determined.

## Introduction

Childhood obesity often persists into adulthood (1), bringing with it a host of metabolic abnormalities (2). Compared to people who develop obesity as adults, those with childhood-onset obesity (CO) face a heightened risk of type 2 diabetes (3–11). While the mechanisms behind this increased risk remain unclear, chronic, low-grade inflammation—implicated in both insulin resistance and β-cell dysfunction (12)—may play a key role. Inflammation also contributes to arterial stiffness, a marker of subclinical cardiovascular disease (13). Increased arterial stiffness often precedes the development of insulin resistance and type 2 diabetes (14, 15) and is linked to the microvascular and macrovascular complications of type 2 diabetes (16).

Adipose tissue serves as a central hub linking inflammation to cardiometabolic disease. As both an immunological and endocrine organ, it harbors diverse immune cells and secretes inflammatory factors that can act locally or systemically. During obesity development in mice, adipose tissue immune cell and secretory profiles shift from anti-inflammatory to pro-inflammatory (17). Children with obesity already exhibit macrophage infiltration in adipose tissue and elevated circulating inflammatory markers, such as C-reactive protein (18). Whether their adipose tissue and systemic inflammation worsen as they enter adulthood remains unknown.

Our group has compared adipose tissue macrophage (ATM) populations between female bariatric surgery patients with CO and adult-onset obesity (AO), matched for type 2 diabetes status. We found that the age of obesity onset did not affect M1-like ‘pro-inflammatory’ and M2-like ‘anti-inflammatory’ macrophage content in abdominal subcutaneous adipose tissue (SAT) and visceral adipose tissue (VAT) (19). However, we do not know if such similarities manifest in adults with milder obesity before the onset of comorbidities or if they extend to other immune cells and circulating adipokines.

Furthermore, we do not fully understand whether conventional weight loss treatments can effectively target inflammation in people with CO and AO. While some pro-inflammatory adipose tissue immune cells decrease after bariatric surgery (20, 21), this is not always the case after lifestyle interventions—despite cardiometabolic improvements (22, 23). We suspect that the age of obesity onset may contribute to this variability.

In this preliminary, hypothesis-generating study, we aimed to examine the effect of age of obesity onset (CO vs. AO) on abdominal and femoral SAT macrophage and T-cell populations (primary outcomes), as well as circulating adipokines and subclinical cardiometabolic risk factors (hyperinsulinemia and arterial stiffness) before and after moderate (~10%) weight loss.

## Materials and methods

### Participants and study design

We recruited healthy, non-smoking adult females (age = 25–40 years; BMI = 30.0–39.9 kg/m^2^) who acquired obesity either pre-/peri-puberty (CO; *n* = 14) or after the age of 18 (AO; *n* = 13). Participants were sedentary or lightly active, weight stable ( ± 2 kg) for at least two months, and free of nicotine use. Individuals were excluded if their medication use (e.g., antidepressants, antihypertensives), past or current medical conditions, or surgical history (e.g., gastric bypass) could affect study outcomes or interfere with participation. Females who were menopausal, pregnant, or breastfeeding were also excluded.

To verify the age of obesity onset, participants provided photographic proof of body size around puberty (~10–14 years old) and verified their body size at different ages using the Collins Childhood Body Rating Scales (24) and the Stunkard Body Rating Scale (25). A subset of these participants was included in a previously published study (26). The study was approved by the Concordia University Human Research Ethics Committee, and all participants provided written informed consent.

The study design has been previously detailed (26). In brief, the study consisted of a baseline 2-week weight stabilization period, a weight loss period, and a final 2-week weight stabilization period. The weight loss period ended when participants lost approximately 10% of their initial body weight. Toward the end of each weight stabilization period, after a 12-hour fast, participants underwent anthropometric, body composition, and arterial stiffness assessments; indirect calorimetry to measure resting energy expenditure (Field Metabolic System and Flow Kit 500, Sable Systems, Las Vegas, NV, USA); blood sampling; and needle-aspirated abdominal (lateral periumbilical region) and femoral (lateral upper thigh) SAT biopsies. Standard clinical blood measurements (serum glucose, total cholesterol, HDL cholesterol, LDL cholesterol, and triglycerides) were only taken at baseline. The biopsy procedure has been previously described (27). Eight participants with CO and six participants with AO completed assessments both before and after weight loss.

### Lifestyle weight loss protocol

The weight loss protocol was adapted from a previously successful intervention (28). Baseline daily energy needs were calculated by multiplying resting energy expenditure by an activity factor of 1.2–1.3, reflecting a sedentary to lightly active lifestyle. Participants were prescribed a 30% energy deficit—achieved through a 20% reduction in energy intake and a 10% increase in energy expenditure. Energy intake goals were met using exchange lists for meal planning (29), with a macronutrient distribution of 50–60% carbohydrate, 20% protein, and 20–30% fat.

To increase energy expenditure, participants completed three 45-minute moderate-to-vigorous intensity treadmill or elliptical sessions per week at the School of Health (SOH, formerly the PERFORM Centre). The sessions were preprogrammed on a Technogym key (Technogym USA Corp., Fairfield, New Jersey, USA), and participants wore heart rate monitors to track intensity. Target heart rate zones were calculated using the Karvonen equation (30), progressing from 40–50% heart rate reserve (HRR) in weeks 1–2, to 50–60% HRR in weeks 3–4, with intervals alternating between 60 and 80% HRR thereafter.

Participants were trained to use the Borg Rating of Perceived Exertion scale (31) to monitor exercise intensity during any sessions performed outside the SOH without a heart rate monitor. Weekly weigh-ins and follow-ups were conducted throughout the intervention.

### Anthropometric and body composition measurements

Height and weight were measured to the nearest 0.1 cm and 0.1 kg, respectively, using a fixed-wall stadiometer (Seca 216, Seca Corp., Chino, CA, USA) and a calibrated scale (DIN 2, AmCells Corp., Vista, CA, USA), with participants wearing light clothing and no shoes. Total and regional body composition were assessed by dual-energy x-ray absorptiometry (DXA; Lunar Prodigy Advance, GE Healthcare, Madison, WI, USA; Encore software version 14.10). When necessary, regions of interest were manually adjusted by trained technicians to ensure consistency across participants.

SAT and VAT areas were quantified from a single-slice (10 mm) computed tomography scan at the L2–L3 level (Revolution Evo, GE Healthcare, Madison, WI, USA) using Slice-O-Matic software (version 5.0; Tomovision, Montréal, QC, Canada). Android SAT and VAT mass were estimated by multiplying the ratio of SAT or VAT to total adipose tissue (TAT) (from CT) by DXA-derived android fat mass (e.g., CT VAT [cm^2^]/CT TAT [cm^2^] × DXA android fat [kg] = android VAT [kg]) (32).

### Subclinical cardiometabolic risk assessments

We assessed plasma insulin concentration and arterial stiffness as markers of subclinical cardiometabolic risk. Plasma insulin concentration was measured by enzyme-linked immunosorbent assay (ELISA) (R&D Systems, Oakville, ON, Canada). Arterial stiffness was assessed as carotid-femoral pulse wave velocity using applanation tonometry (SphygmoCor, AtCor Medical, Sydney, Australia) as previously described (33).

### Circulating adipokines

Plasma concentrations of plasminogen activator inhibitor-1 (PAI-1), monocyte chemoattractant protein-1 (MCP-1), and resistin were measured using a Human ProcartaPlex™ Simplex Kit (Thermo Fisher Scientific [Invitrogen], Waltham, MA, USA). Plasma concentrations of leptin, adiponectin, interleukin (IL)-8 (R&D Systems, Oakville, ON, Canada), and IL-6 (Abcam, Toronto, ON, Canada) were measured by ELISA. Adiponectin is an anti-inflammatory, insulin-sensitizing adipokine, whereas the others are pro-inflammatory.

### SAT immune cell analysis

SAT immune cells were isolated and analyzed by flow cytometry using a validated protocol developed by our lab (27). Briefly, the stromovascular cells from approximately 1 g of SAT were isolated by collagenase digestion, purified, stained with CD68, CD206, CD3, CD4, and CD8 antibodies (**Supplementary Table S1**), and analyzed using an 8-color BD FACSVerse (BD Bioscienes, San Jose, California, USA) and FlowJo software version 9.3.2 (Treestar Inc., Ashland, Oregon, USA). **Supplementary Table S2** shows our single-stain and fluorescence-minus-one controls. We quantified the number of CD68^+^CD206^-^ (M1-like) macrophages, CD68^+^CD206^+^ (M2-like) macrophages, CD3^+^CD4^+^ (T helper or T regulatory) cells, and CD3^+^CD8^+^ T cells. Our gating strategy for immune cell identification is displayed in **Supplementary Figure S1**. We expressed immune cell quantities as a percentage of live stromovascular cells.

### Complementary adipose tissue analyses

We measured mean adipocyte volume using the collagenase digestion method, as previously described (34). Isolated adipocytes were imaged with phase-contrast microscopy (Motic AE2000 TRI, Motic [Xiamen] Electric Group Co., Ltd., Xiamen, China). The cross-sectional areas of 100 randomly selected adipocytes were measured using FIJI software (35) and converted to volumes assuming a spherical shape. An example microscopy image is shown in **Supplementary Figure S2**.

To measure SAT-secreted adiponectin, SAT explants were cultured *ex vivo* in Medium 199 supplemented with insulin, dexamethasone, antibiotics, and NaHCO_3_ (3 mL per 100 mg SAT). After 24 hours, the medium was replaced with fresh medium lacking insulin and dexamethasone. The conditioned medium was collected after an additional 24 hours, and adiponectin concentration was measured by ELISA (R&D Systems, Oakville, ON, Canada).

### Statistical analyses

Data analyses were conducted using SAS version 9.4 (SAS Institute Inc., Cary, NC, USA). Baseline participant characteristics were reported as mean (SEM) and compared between groups with independent t-tests.

For our main analyses, we used marginal models (SAS PROC MIXED with the REPEATED statement) and applied restricted maximum likelihood estimation to handle unbalanced data and adjust the residuals’ covariance structure flexibly. The models included the adipose tissue immune cell percentages, adipocyte size, or SAT-secreted adiponectin as outcomes and group (obesity onset), SAT region, time (weight loss), and all two‐way and three‐way interactions as fixed factors. The models for circulating adipokines included group, time, and the group-by‐time interaction as fixed factors. We used likelihood ratio χ² tests to select the appropriate covariance structure (compound symmetry, compound symmetry heterogeneous, or unstructured) for each model. We assessed the normality of residuals for each model using the Shapiro-Wilk test and visual inspection, and natural log-transformed dependent variables when necessary. The Kenward–Roger method was used to estimate the degrees of freedom (36). We decomposed significant interactions graphically and with relevant within‐ and between‐group contrasts. When the three‐way interaction was significant, we tested the group-by‐time and group‐by‐region interactions at each level of the third variable (region and time, respectively) and examined simple contrasts as needed.

We expressed model results as least-squares means (lsmeans) (95% CI) or differences in lsmeans (95% CI). The lsmeans from transformed data were back‐transformed to the original scale for easier interpretation. The differences in lsmeans were back‐transformed for logged outcomes, giving the ratio of the geometric lsmeans (37) or fold difference. We computed Cohen’s *d* (*d* = 2*t*(sqrt(*df*))) using the model t values and degrees of freedom (*df*) (38), and then converted it to Hedges' *g* (*g* = *d*(1 − 3/(4*df* − 1)) to adjust for small sample size bias (39). Statistical significance was set at *p* < 0.1 for interactions and *p* < 0.05 for main effects and contrasts, consistent with prior research (40, 41).

## Results

### Participant characteristics

At baseline, participants with CO and AO did not differ in mean age, BMI, or clinical blood measurements (**Table 1**). There were no differences in baseline characteristics between participants who completed the weight loss protocol and those who did not (data not shown).

**Table 1:** Demographic and clinical characteristics of study participants.

Characteristic	Childhood-onset obesity (*n* = 14)	Adult-onset obesity (*n* = 13)	*p* value
Age (years)	30.2 (1.0)	30.9 (0.8)	0.583
Body Mass Index (kg/m^2^)	33.5 (0.8)	33.9 (0.8)	0.732
Glucose (mmol/L)	4.6 (0.1)^a^	4.7 (0.1)	0.663
Triglycerides (mmol/L)	1.0 (0.1)	1.2 (0.1)	0.405
Total Cholesterol (mmol/L)	4.5 (0.2)	4.4 (0.2)	0.961
HDL Cholesterol (mmol/L)	1.3 (0.1)	1.2 (0.1)	0.616
LDL Cholesterol (mmol/L)	2.7 (0.2)	2.6 (0.2)	0.879

Results are means (SEM).

a: *n* = 13.

HDL, high-density lipoprotein; LDL, low-density lipoprotein.

Participants lost an average of 8 kg, or 9 % of their initial weight. Total and percent body fat, as well as android VAT and SAT, did not differ between obesity-onset groups at baseline and declined similarly across groups with weight loss. The android-to-gynoid fat ratio remained unchanged with weight loss in both groups (**Table 2**).

**Table 2:** Body weight and composition before and after weight loss.

Characteristic	Baseline		Final		Effect, *p* value		
	Childhood-onset obesity (*n* = 14)	Adult-onset obesity (*n* = 13)	Childhood-onset obesity (*n* = 8)	Adult-onset obesity (*n* = 6)	Group	Time	Group x Time
Weight (kg)	91.3 (85.9, 96.6)	94.0 (88.5, 99.5)	82.6 (77.1, 88.2)	85.9 (80.0, 91.8)	0.428	<0.001	0.806
Total Body Fat (kg)	41.1 (37.3, 44.8)	42.7 (38.7, 46.6)	33.4 (29.8, 37.0)	37.5 (33.4, 41.7)	0.176	0.004	0.402
Total Body Fat (%)	45.2 (42.9, 47.5)	45.4 (43.1, 47.8)	41.7 (39.4, 44.1)	42.4 (39.9, 45.0)	0.768	<0.001	0.619
Android VAT (kg)	0.79 (0.56, 1.0)	0.82 (0.56, 1.1)	0.67 (0.43, 0.91)	0.61 (0.36, 0.86)	0.921	0.003	0.332
Android SAT (kg)	2.9 (2.6, 3.3)	2.8 (2.5, 3.2)	2.4 (2.0, 2.7)	2.4 (2.1, 2.8)	0.903	<0.001	0.173
Android Fat (kg)/Gynoid Fat (kg)	0.54 (0.48, 0.60)	0.47 (0.41, 0.53)	0.53 (0.45, 0.60)	0.48 (0.39, 0.56)	0.196	0.780	0.552

Results are least squares means (95 % CI).

SAT, subcutaneous adipose tissue; VAT, visceral adipose tissue.

### Subclinical cardiometabolic risk factors

**Figure 1:**
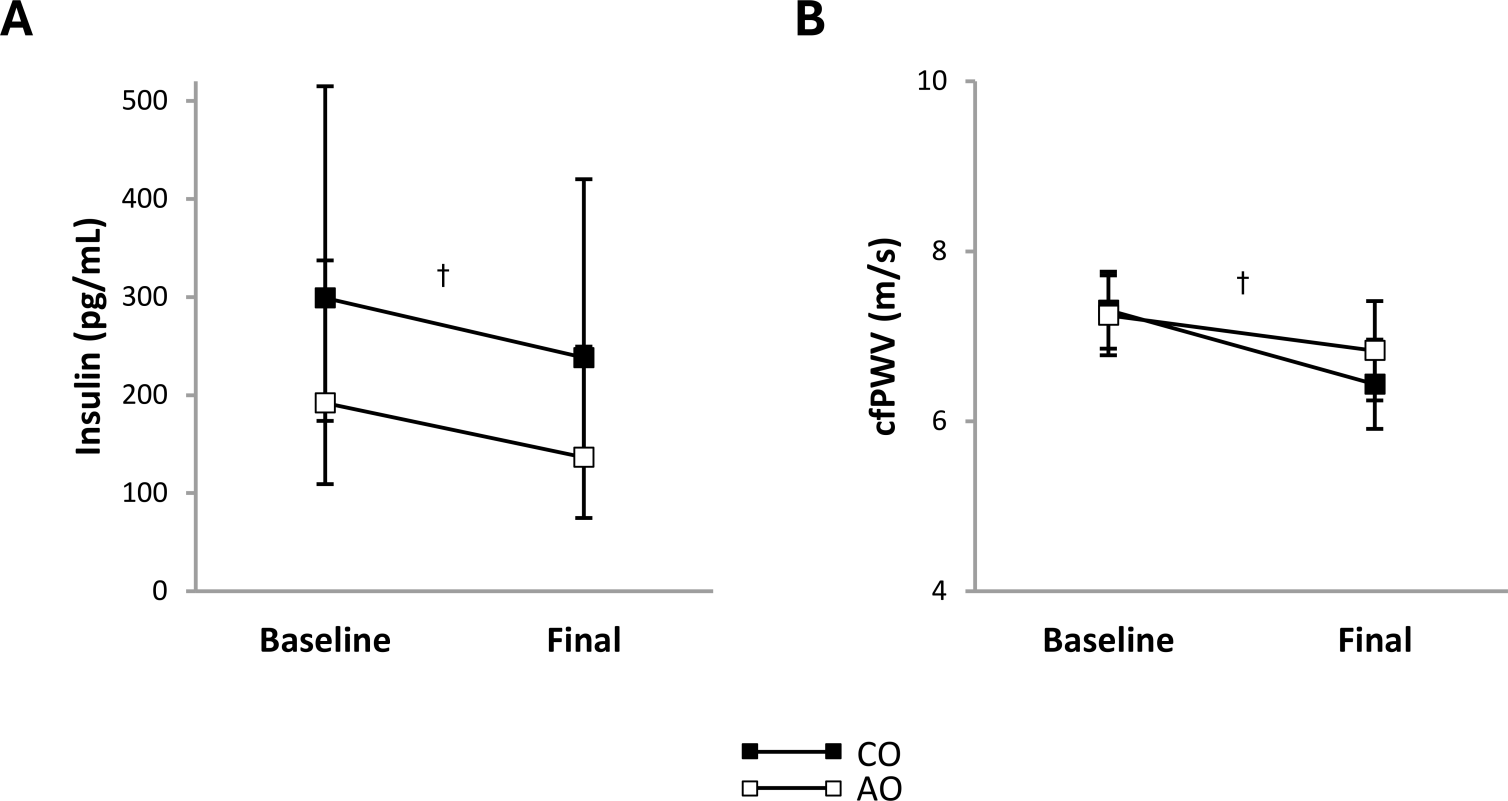
Subclinical cardiometabolic risk factors in females with childhood-onset and adult-onset obesity before and after moderate weight loss. **(A, B)** Subclinical cardiometabolic risk factors by group over time. The outcome was natural log-transformed in **(A)** prior to analysis but is displayed as back-transformed values. Results are presented as least-squares means (95% CI). In **(A)**, ^†^time, *p* = 0.016: baseline > final. In **(B)**, ^†^time, *p* = 0.001: baseline > final. cfPWV, carotid-femoral pulse wave velocity (arterial stiffness); AO, group with adult-onset obesity; CO, group with childhood-onset obesity.

The obesity-onset groups did not differ in plasma insulin concentration or arterial stiffness at baseline. Across groups, plasma insulin concentration decreased 1.33-fold (95% CI: 1.07, 1.66; *p* = 0.016; *g* = 1.50) and arterial stiffness decreased by 0.64 m/s (95% CI: 0.30, 0.99; *p* = 0.001; *g* = 1.98) (**Figure 1**).

### Adipokines

**Figure 2:**
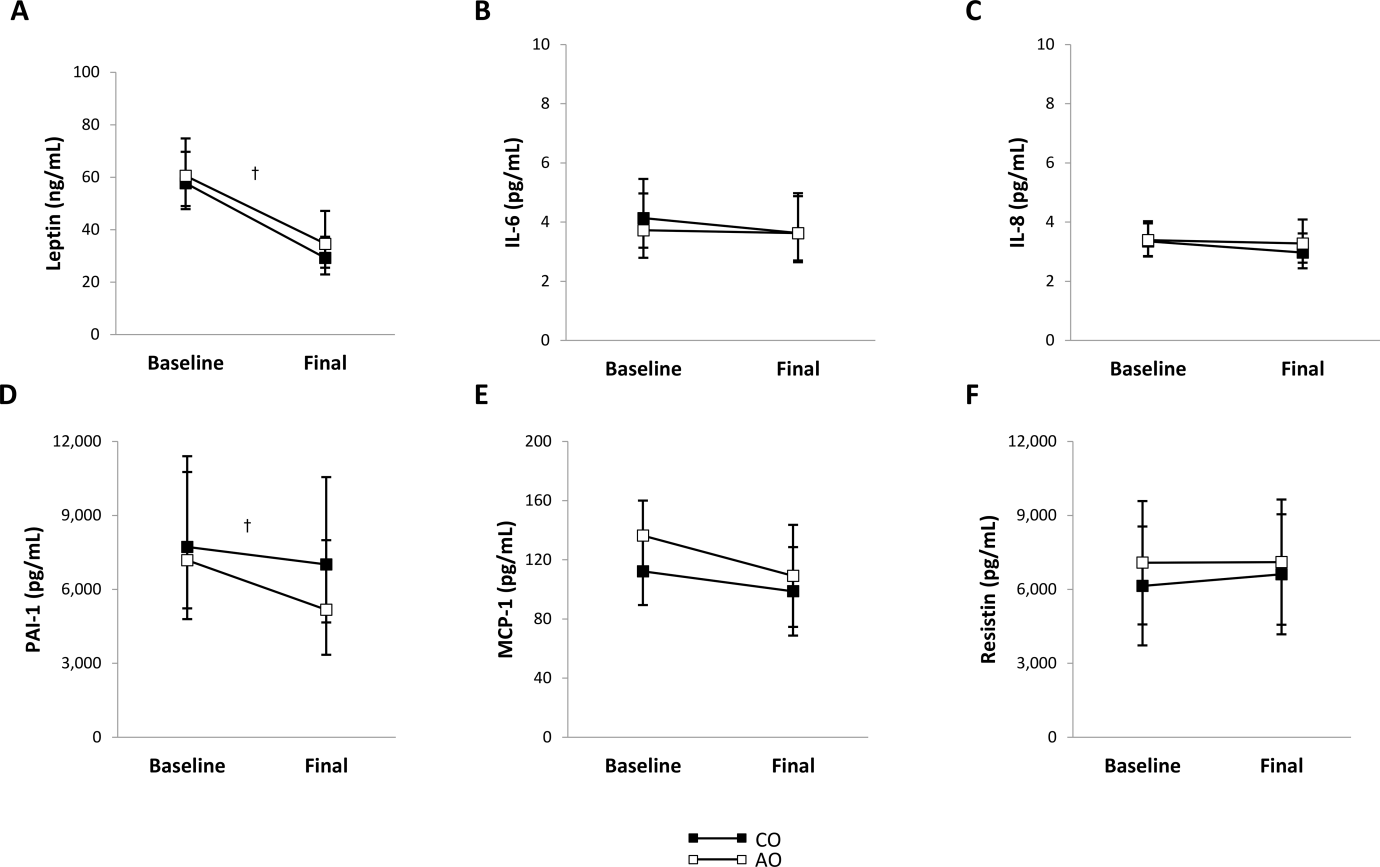
Plasma proinflammatory adipokine concentrations in females with childhood-onset and adult-onset obesity before and after moderate weight loss. **(A–F)** Plasma proinflammatory adipokine concentrations by group over time. The outcomes were natural log-transformed in (A–D) prior to analysis but are displayed as back-transformed values. Results are presented as least-squares means (95% CI). In **(A)**, ^†^time, *p* < 0.001: baseline > final. In **(D)**, ^†^time, *p* = 0.016: baseline > final. AO, group with adult-onset obesity; CO, group with childhood-onset obesity; IL, interleukin; MCP-1, monocyte chemoattractant protein-1; PAI-1, plasminogen activator inhibitor-1.

**Figure 3:**
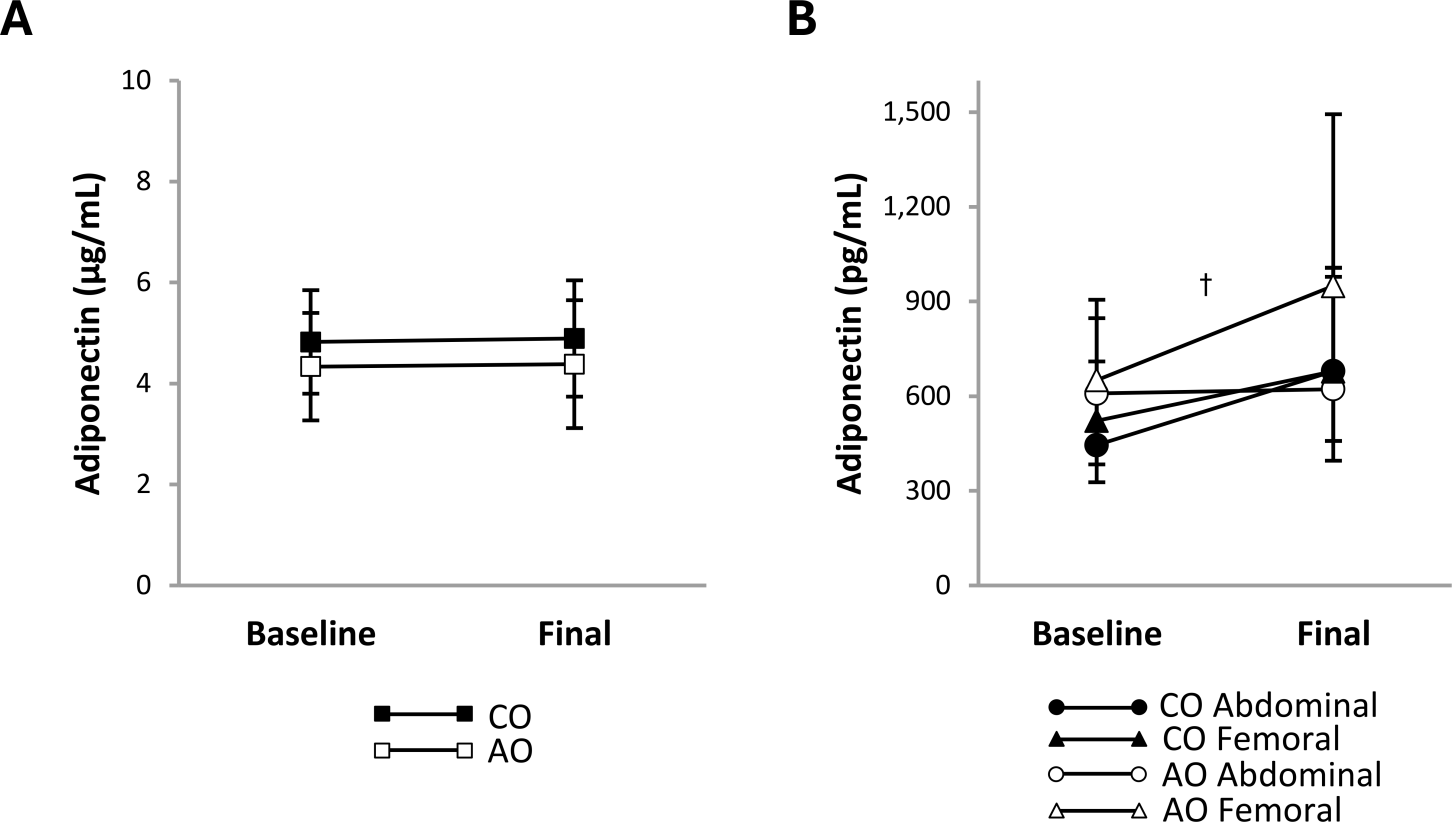
Adiponectin concentrations in plasma and regional subcutaneous adipose tissue-conditioned media from females with childhood-onset and adult-onset obesity before and after moderate weight loss. Adiponectin concentration in plasma **(A)** and subcutaneous adipose tissue-conditioned media **(B)** by group over time. The outcome was natural log-transformed in **(B)** prior to analysis but is displayed as back-transformed values. Results are presented as least-squares means (95% CI). In **(B)**, ^†^time, *p* = 0.032: baseline < final. AO, group with adult-onset obesity; CO, group with childhood-onset obesity.

After weight loss, plasma leptin concentration decreased 1.86-fold (95% CI: 1.50, 2.30; *p <* 0.001; *g* = 2.77) and plasma PAI-1 concentration decreased 1.24-fold (95% CI: 1.05, 1.46; *p* = 0.016; *g* = 1.46) across groups (**Figures 2A, D**). There were no group, time, or group-by-time interaction effects on the plasma concentrations of other proinflammatory adipokines (**Figures 2B, C, E, F**) or adiponectin (**Figure 3A**). The adiponectin concentration in SAT-conditioned media, however, increased after weight loss (1.31-fold (95% CI: 1.02, 1.68); *p* = 0.032; *g* = 0.56) across groups and SAT regions (**Figure 3B**).

### Adipocyte size

**Figure 4:**
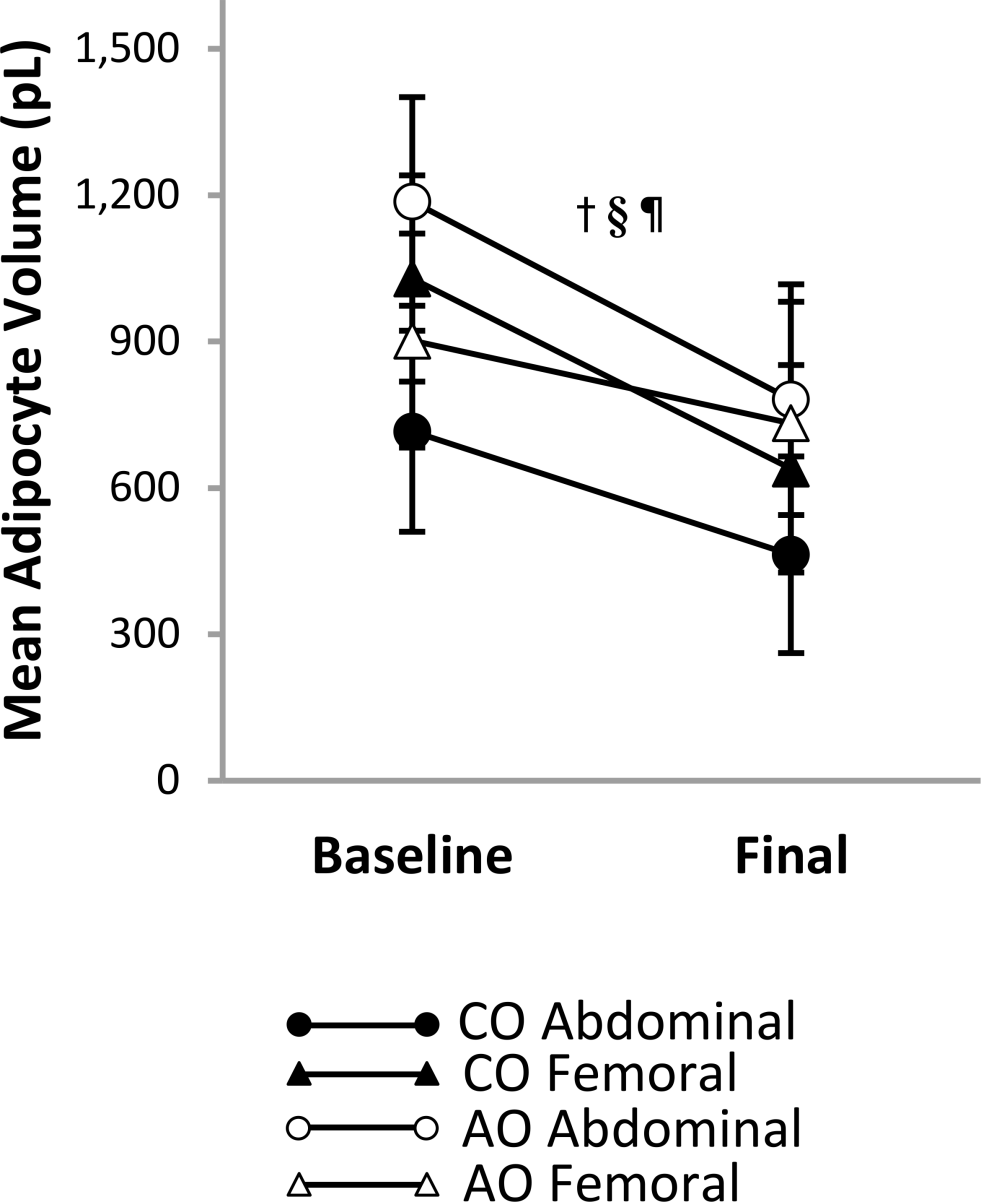
Regional subcutaneous adipocyte size in females with childhood-onset and adult-onset obesity before and after moderate weight loss. Adipocyte size by group and subcutaneous adipose tissue region over time. Results are presented as least-squares means (95% CI). ^†^time, *p* = 0.005; ^§^region-by-onset, *p* < 0.001; ^¶^group-by-region-by-time, *p* = 0.004: group-by-time in Abdominal region, *p* = 0.454 (across time: CO < AO, *p* = 0.001; across groups: baseline > final, *p* = 0.004); group-by-time in Femoral region, *p* = 0.331 (across time: CO = AO, *p* = 0.867; across groups: baseline > final, *p* = 0.021); group-by-region at baseline, *p* < 0.001 (CO: Abdominal < Femoral, *p* = 0.002; AO: Abdominal > Femoral, *p* = 0.005); group-by-region at final, *p* = 0.049 (CO: Abdominal < Femoral, *p* = 0.021; AO: Abdominal = Femoral, *p* = 0.547). AO, group with adult-onset obesity; CO, group with childhood-onset obesity.

There was a group-by-region-by-time interaction on mean adipocyte size (**Figure 4**). The group-by-region interaction was significant at baseline; mean adipocyte size was greater in the femoral region than in the abdominal region in the CO group (313 pL [95% CI: 129, 497]; *p* = 0.002; *g* = 1.36) but greater in the abdominal region than in the femoral region in the AO group (285 pL [95% CI: 95, 476]; *p* = 0.005; *g* = 1.19). The group-by-time interaction was not significant in the abdominal or femoral region. Across time, mean adipocyte size was 394 pL (95% CI: 180, 609) smaller in the CO group than in the AO group in the abdominal region (*p* = 0.001; *g* = -1.68) and not different between groups in the femoral region. Across groups, adipocyte size decreased with weight loss by 329 pL (95% CI: 120, 539) in the abdominal region (*p* = 0.004; *g* = -1.43) and by 280 pL (95% CI: 48, 512) in the femoral region (*p* = 0.021; *g* = -1.16). A slight difference in the change over time between regions in the AO group (larger decrease in the abdominal region) led to a group-by-region interaction after weight loss that manifested differently than at baseline. While adipocyte size remained greater in the femoral region than in the abdominal region in the CO group (176 pL [95% CI: 31, 321]; *p* = 0.021; *g* = 1.43), there was no regional difference in the AO group after weight loss.

### SAT immune cells

**Figure 5:**
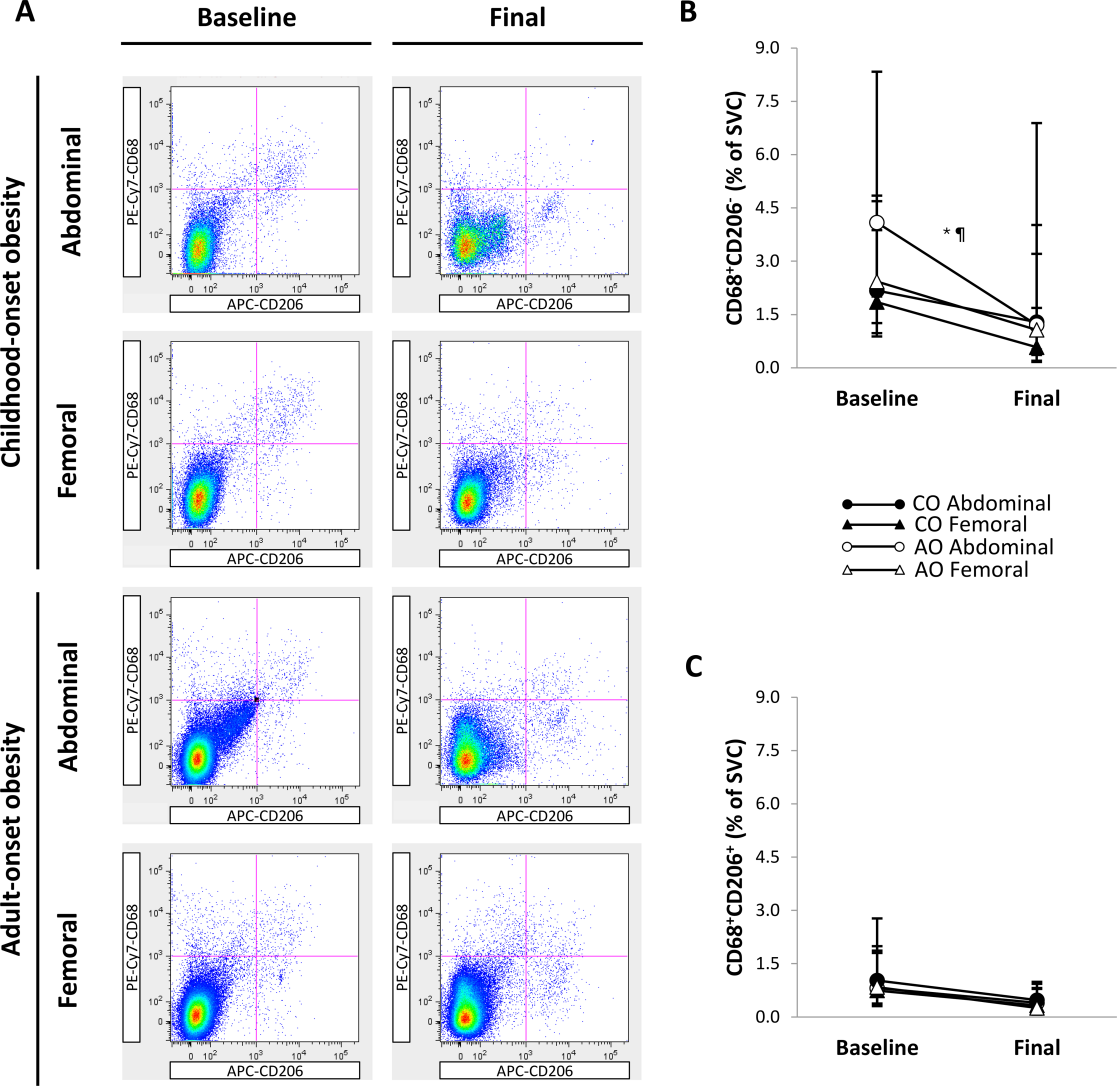
Regional subcutaneous adipose tissue macrophage populations in females with childhood-onset and adult-onset obesity before and after moderate weight loss. **(A)** Representative flow cytometry plots of macrophage populations. In each plot, the top left quadrant shows CD68^+^CD206^-^ macrophages, and the top right quadrant shows CD68^+^CD206^+^ macrophages. **(B, C)** Macrophage populations by group and subcutaneous adipose tissue region over time. Outcomes were natural log-transformed prior to analysis but are displayed as back-transformed values. Results are presented as least-squares means (95% CI). In **(B)**, ^*^region, *p* = 0.024; ^¶^group-by-region-by-time, *p* = 0.076: group-by-time in Abdominal region, *p* = 0.402 (across time: CO = AO, *p* = 0.537; across groups: baseline > final, *p* = 0.045); group-by-time in Femoral region, *p* = 0.748 (across time: CO = AO, *p* = 0.428; across groups: baseline = final, *p* = 0.076); group-by-region at baseline, *p* = 0.166 (across groups: Abdominal > Femoral, *p* = 0.013); group-by-region at final, *p* = 0.215 (across groups: Abdominal = Femoral, *p* = 0.106). Although these two-way interactions cannot fully explain the three-way interaction, simple contrasts indicated that the regional difference (Abdominal > Femoral) at baseline and the change in the Abdominal region (baseline > final) occurred in AO and not in CO. These contrasts correspond with the graphical depiction showing that the change over time was less parallel for CO and AO in the Abdominal region compared to the Femoral region. AO, group with adult-onset obesity; CO, group with childhood-onset obesity; SVC = stromovascular cells.

**Figure 5A** shows representative flow cytometry plots of macrophage populations by group, SAT region, and time. There was a group-by-region-by-time interaction on the percentage of M1-like CD68^+^CD206^-^ macrophages (**Figure 5B**), indicating that the group difference in the change over time was not consistent across SAT regions, or that the group difference in regional SAT variation was not consistent before and after weight loss. However, the group-by-region interaction before and after weight loss and the group-by-time interaction in the abdominal and femoral regions were not significantly different from zero, suggesting that these two-way interactions did not fully explain the three-way interaction. Across time, the percentage of CD68^+^CD206^-^ macrophages was not significantly different between the CO and AO groups in the abdominal and femoral regions. Across groups, the percentage of CD68^+^CD206^-^ macrophages was 1.40-fold (95% CI: 1.08, 1.83; *p* = 0.013; *g* = 1.20) greater in the abdominal region than in the femoral region at baseline and declined in the abdominal region (2.40-fold [95% CI: 1.02, 5.62]; *p* = 0.045; *g* = 1.00) but not in the femoral region with weight loss. As a result, there was no regional difference across groups post-weight loss. Simple contrasts, however, indicated that the regional difference at baseline and the decline in the abdominal region with weight loss were specific to the AO group. These contrasts help explain the three-way interaction that can be depicted on the graph: the change over time was less parallel for the CO and AO groups in the abdominal region (higher at baseline and larger decrease in the AO group compared to the CO group, though not significant) than in the femoral region; and the regional difference was more pronounced in the AO group than in the CO group before weight loss, but not after weight loss. There were no group, region, time, or interaction effects on the percentage of M2-like CD68^+^CD206^+^ macrophages (**Figure 5C**).

**Figure 6:**
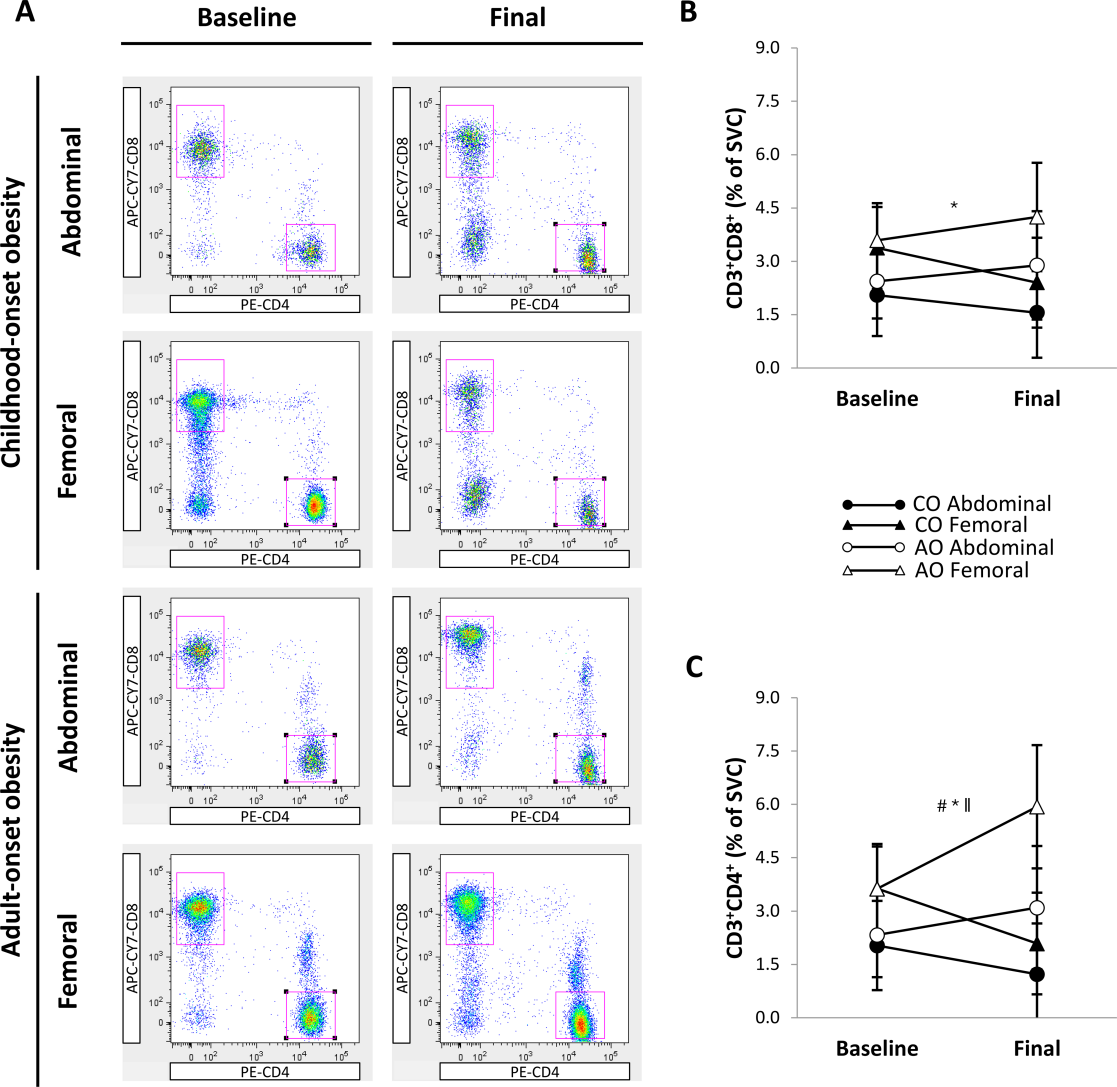
Regional subcutaneous adipose tissue T-cell populations in females with childhood-onset and adult-onset obesity before and after moderate weight loss. **(A)** Representative flow cytometry plots of T-cell populations. In each plot, the top left gate shows CD3^+^CD8^+^ T cells, and the lower right gate shows CD3^+^CD4^+^ T cells. **(B, C)** T-cell populations by group and subcutaneous adipose tissue region over time. Results are presented as least-squares means (95% CI). In **(B)**, ^*^region, *p* = 0.001: Abdominal < Femoral. In **(C)**, ^#^group, *p* = 0.043; ^*^region, *p* < 0.001: Abdominal < Femoral; ^∥^group-by-time, *p* = 0.004 (across regions at baseline: CO = AO, *p* = 0.843; across regions in CO: baseline = final, *p* = 0.063; across regions in AO: baseline < final, *p* = 0.025; across regions at final: CO < AO, *p* = 0.004). AO, group with adult-onset obesity; CO, group with childhood-onset obesity; SVC, stromovascular cells.

**Figure 6A** shows representative flow cytometry plots of T-cell populations by group, SAT region, and time. There was a region effect but no group, time, or interaction effects on the percentage of CD3^+^CD8^+^ T cells (**Figure 6B**). The percentage of CD3^+^CD8^+^ T cells was greater in the femoral region than in the abdominal region across groups and time (1.17 percentage points [95% CI: 0.48, 1.86]; *p* = 0.001; *g* = 1.07). Similarly, the percentage of CD3^+^CD4^+^ T cells (**Figure 6C**) was greater in the femoral region than in the abdominal region across groups and time (1.65 percentage points [95% CI: 0.87, 2.43]; region effect, *p* < 0.001; *g* = 1.32). In addition, there was a group-by-time interaction on the percentage of CD3^+^CD4^+^ T cells. Across regions, the percentage of CD3^+^CD4^+^ T cells did not differ between groups pre-weight loss and increased after weight loss in the AO group only (1.54 percentage points [95% CI: 0.20, 2.87]; *p* = 0.025; *g* = 0.64). Therefore, the percentage of CD3^+^CD4^+^ T cells was higher in the AO group than in the CO group across regions post-weight loss (2.86 percentage points [95% CI: 0.99, 4.73]; *p* = 0.004; *g* = 0.85).

## Discussion

This study was motivated by the increased risk of type 2 diabetes associated with CO and is the first of its kind to examine the effects of age of obesity onset on regional SAT immune cells and systemic inflammation before and after weight loss. Contrary to our expectations, we did not find a more pro-inflammatory SAT immune cell profile in females with CO than in those with AO. Instead, we found a slightly higher percentage of M1-like macrophages in the abdominal SAT of females with AO, and no other differences in macrophage or T-cell percentages between groups. Fasting plasma adipokine and insulin concentrations, as well as arterial stiffness, were also not significantly different between groups. Moderate (~10%) weight loss similarly reduced arterial stiffness and plasma concentrations of insulin, leptin, and PAI-1 in females with CO and AO. However, SAT immune cell percentages only shifted in females with AO. After weight loss, their M1-like macrophages in abdominal SAT decreased, while their CD3^+^CD4^+^ T cells in abdominal and femoral SAT increased.

Consistent with our baseline findings on M1-like macrophages, females with AO exhibit greater IL-6 gene expression in abdominal SAT than do those with CO (42). A mouse study by Wernstedt Asterholm et al. (43) prompts us to question whether increased abdominal SAT pro-inflammatory macrophages in AO represent a temporary adaptive response to recent weight gain rather than a driver of chronic inflammation. Wernstedt Asterholm et al. (43) found that, during the early stages of obesity development in mice, adipose tissue inflammation is crucial for proper extracellular matrix remodeling and angiogenesis, processes that promote adipogenesis. They postulated that by promoting healthy tissue expansion, acute adipose tissue inflammation can resolve before becoming chronic (43). While no studies have compared adipogenic capacity between people with CO and AO, our lab has provided evidence suggesting increased lipogenic capacity in females with AO (42).

Although it is appealing to view the increased abdominal SAT proinflammatory macrophages in AO as an adaptive response to recent weight gain, we must consider that adult obesity typically develops over several months or even years in humans. This raises questions about what constitutes an ‘acute’ inflammatory response to weight gain in humans and how long healthy adipose tissue remodeling can persist before chronic, low-grade inflammation takes over. Short-term overfeeding studies in healthy adults show that after a ~3-kg weight gain, the total number of macrophages in abdominal SAT remains stable. However, the M1/M2 macrophage ratio increases along with markers of extracellular matrix remodeling and angiogenesis (44, 45). What remains unclear is whether these changes after weight gain are tempered once weight stabilizes over the long term. In our study, participants were weight-stable for at least three months, yet we still observed slight differences in abdominal SAT M1-like macrophages between those with recent weight gain (AO) and those with CO. Studies comparing bariatric surgery patients with CO and AO indicate that by the time they have severe obesity with comorbidities, their ATM profiles converge (19). The adiposity trajectories leading to this convergence remain to be studied.

Even though our findings suggest that SAT immune cells and circulating adipokines may not explain the increased type 2 diabetes risk associated with CO, they do not discount the role of inflammation entirely. While the degree of SAT and systemic inflammation is one consideration for cardiometabolic risk, the duration of exposure to inflammation is another. Still, this reasoning does not align with our subclinical findings. If those with CO experience chronic, low-grade inflammation for longer, why don’t they exhibit worse arterial stiffness than those with AO? Differences in other triggers of arterial stiffness, such as oxidative stress, endothelial dysfunction, or structural changes in the artery, could provide an explanation.

Average plasma insulin concentration was ~100 pmol/L greater in females with CO than in those with AO, and although this difference was not statistically significant, it may still be clinically relevant. Hyperinsulinemia, a well-established risk factor for type 2 diabetes, remains difficult to define due to variability in insulin assays. However, the mean plasma insulin concentrations of ~300 pmol/L in our CO group and ~200 pmol/L in our AO group are notably higher than the ~60 pmol/L we measured in a small sample of lean females using the same methodology. Plasma insulin concentrations reflect the balance between insulin secretion and clearance. An increase in the former and a decrease in the latter can lead to fasting and postprandial hyperinsulinemia in people with obesity (46).

Drawing from current evidence, we suspect that compensatory hyperinsulinemia may be more common in AO, whereas hypersecretion-induced hyperinsulinemia may be more common in CO. Studies using the hyperinsulinemic-euglycemic clamp technique have found worse insulin resistance in people with AO compared to those with CO (47, 48). Moreover, at the same level of glycemia and insulin sensitivity, children with obesity have hyperresponsive β-cells compared to adults with obesity (49–51). This hyperresponsiveness could lead to accelerated β-cell exhaustion and an earlier onset of type 2 diabetes in adults with CO (52). Studies comparing insulin kinetics and multi-organ insulin sensitivity between adults with CO and AO over time are required to test this hypothesis.

Many cardiometabolic risk factors improve following lifestyle weight loss interventions (53–55), but whether changes in adipose tissue immune cell profiles drive these improvements remains unclear. Collectively, human studies suggest that total abdominal SAT macrophage content increases or remains stable in the early phases of rapid or gradual diet and/or exercise-induced weight loss (< ~10%), despite improvements in cardiometabolic risk factors (22, 23, 55–59). However, with continued weight loss (> ~15%), total abdominal SAT macrophage content decreases (55, 56, 60). There is also evidence that exercise, with or without weight loss, shifts abdominal SAT macrophages toward an anti-inflammatory M2-like phenotype (57, 61). In our study, only females with AO experienced a decrease in the proportion of abdominal SAT M1-like macrophages after weight loss, eliminating the baseline difference between those with AO and CO. Therefore, in some people, a 10% weight loss achieved through diet and exercise may reduce pro-inflammatory macrophages, with baseline levels possibly playing a role.

In our study, the percentages of abdominal and femoral SAT CD3^+^CD8^+^ T cells (pro-inflammatory) did not change after weight loss, regardless of obesity onset. In contrast, the proportion of CD3^+^CD4^+^ T cells increased in both SAT regions in females with AO, but not in those with CO. Our findings in females with AO align with those of Kratz et al., who examined changes in abdominal SAT T-cell populations after a 1-year lifestyle intervention (~7% weight loss) in people with obesity and type 2 diabetes (23). Given their participants were older (~50 years), it is plausible that a higher proportion had AO.

SAT Treg cells, an anti-inflammatory subset of CD3^+^CD4^+^ T cells, are both reduced and less functional in adults with obesity (62). Due to chronic antigen stimulation, they adopt an exhausted phenotype marked by impaired activity and a diminished ability to proliferate (62). Interestingly, Cottom et al. found that adipose tissue Treg cells remain low and maintain an exhausted phenotype after weight loss in obese mice (63). If the increase in CD3^+^CD4^+^ T cells in people with AO is due to a rise in Treg cells, it might suggest that their baseline Treg cells—even if similar in quantity to the CO group—are less exhausted. Conversely, in those with CO, 10% weight loss may not be enough to recover Treg cell exhaustion. Further phenotyping of CD3^+^CD4^+^ T cells in people with CO and AO, both before and after weight loss, is needed to explore this possibility.

For people with obesity engaged in lifestyle interventions, an initial weight loss target of 5-10% is considered clinically significant. We found that after 10% weight loss, females with CO and AO improved their plasma insulin concentration and arterial stiffness. However, our findings, along with those of others (22, 55), suggest that 10% weight loss may not be sufficient to induce positive changes in certain aspects of SAT biology—at least in some people. Consistent with other studies (55, 64), we found that circulating concentrations of only select adipokines, leptin and PAI-1, declined in both groups after moderate weight loss. We also found that adipocyte size decreased and adiponectin secreted from SAT increased in both groups, whereas SAT immune cell profiles only changed in those with AO.

While these results may suggest that changes in SAT immune cell profiles are not required for short-term improvements in cardiometabolic risk factors, we wonder whether they are necessary to sustain improvements over the long-term. Since immune cell infiltration is one of the first changes with weight gain, could it be the last to resolve with weight loss before lasting metabolic health is restored? In mice with diet-induced obesity, a proinflammatory immune cell profile in adipose tissue persisted after weight loss and, along with glucose tolerance, worsened after weight regain (63). The authors speculated that a ‘memory-like immunological imprinting’ may contribute to the exacerbated metabolic dysfunction associated with weight regain (63). Interestingly, after a 10% diet-induced weight loss in humans, the lower the reduction in gene expression of immune cell integrins in abdominal SAT, the greater the weight regain over the following nine months (65). These findings may be particularly relevant to those with CO, whose SAT immune cell profile did not change after 10% weight loss and whose hyperplastic abdominal SAT may predispose them to weight regain (66). Longitudinal studies are required to determine whether those with CO need greater weight loss or alternative interventions compared to those with AO to prevent weight regain and sustain metabolic improvements. GLP-1 receptor agonists may offer one such alternative, as they can reduce appetite and—at least in preclinical models—modulate adipose tissue inflammation (67).

We acknowledge that the M1/M2 classification may oversimplify the inflammatory and functional diversity of ATM. Measuring cytokine production from isolated macrophage populations would have provided greater insight into their inflammatory phenotype. We were also unable to assess CD3^+^CD4^+^ T-cell subtypes, which limits our understanding of how they contribute to the SAT inflammatory milieux. Moreover, our analysis was limited to macrophages and T cells in SAT, preventing a comprehensive understanding of the adipose tissue immune landscape. Our findings are derived from a small sample of healthy females with obesity, which limits generalizability to other populations, including males and people with varying degrees of cardiometabolic risk.

A major strength of our study is the use of the gold standard technique, flow cytometry, to quantify immune cell percentages in two SAT regions. SAT immune cell percentages quantified by flow cytometry demonstrate good test-retest reliability (68), which is crucial for our pre-post design. Another strength is that we verified the age of obesity onset through photographic evidence and body rating scales, ensuring accurate classification of participants. Furthermore, our narrow eligibility criteria resulted in groups well-matched for age, body composition, and body fat distribution, enhancing our ability to isolate the effect of age of obesity onset on SAT immune cell profiles.

Our findings provide preliminary evidence that the age of obesity onset influences changes in SAT macrophages and T cells following moderate weight loss. Unexpectedly, females with AO had slightly more proinflammatory macrophages at baseline in abdominal SAT compared to those with CO, though this difference diminished after weight loss. In contrast, T-cell populations in both abdominal and femoral SAT were unaffected by the age of obesity onset at baseline; however, CD3^+^CD4^+^ T cells increased after weight loss only in those with AO. Our results suggest that SAT immune cell profiles may not fully account for the elevated type 2 diabetes risk in people with CO or the short-term cardiometabolic benefits of weight loss. However, the long-term implications of SAT immune changes—or lack thereof—after weight loss remain unclear. Future research should determine if sustained metabolic improvements in people with CO and AO require specific changes in SAT immune cell populations and whether targeted interventions are needed to support these changes.

## Data Availability

The datasets presented in this article are not readily available because ethical approval is first required. Requests to access the datasets should be directed to Sylvia Santosa, s.santosa@concordia.ca.
