# Supplementary material for: The age of obesity onset affects changes in subcutaneous adipose tissue macrophages and T cells after weight loss
Source: Front Immunol. 2025 Aug 4;16:1601847. doi: 10.3389/fimmu.2025.1601847 (PMC12358274; doi:10.3389/fimmu.2025.1601847)
Supplement: Supplementary file 1 [file DataSheet1.pdf]

**Supplementary information to the article "The age of obesity onset affects changes in subcutaneous adipose tissue macrophages and T cells after weight loss"**

**Table S1. Antibody and fluorochrome pairings with supplier and clone information**

| Antibody* | Fluorochrome | Supplier | Clone | Titrated concentration (µl/$10^6$ cells) |
|---|---|---|---|---|
| CD4 | PE | BioLegend | SK3 | 1.5 |
| CD68 | PE-Cy7 | eBioscience | 815CU17 | 1.5 |
| CD206 | APC | eBioscience | 19.2 | 1.5 |
| CD8 | APC-Cy7 | BioLegend | SK1 | 1.5 |
| CD3 | BV510 | BioLegend | SK7 | 1.5 |

*Antibodies were titrated on human adipose tissue to determine the optimal staining volume per $10^6$ cells.

**Table S2. Single stain (SS) cocktails and fluorescence minus one (FMO) controls for flow cytometry experiment**

| | PE | PE-Cy7 | APC | APC-Cy7 | BV510 | Compensation Beads |
|---|---|---|---|---|---|---|
| SS PE | CD4 | | | | | Positive/ negative |
| SS PE-Cy7 | | CD68 | | | | Positive/ negative |
| SS APC | | | CD206 | | | Positive/ negative |
| SS APC-Cy7 | | | | CD8 | | Positive/ negative |
| SS BV510 | | | | | CD3 | Positive/ negative |
| FMO PE | | CD68 | CD206 | CD8 | CD3 | |
| FMO PE-Cy7 | CD4 | | CD206 | CD8 | CD3 | |
| FMO APC | CD4 | CD68 | | CD8 | CD3 | |
| FMO APC-Cy7 | CD4 | CD68 | CD206 | | CD3 | |
| FMO BV510 | CD4 | CD68 | CD206 | CD8 | | |

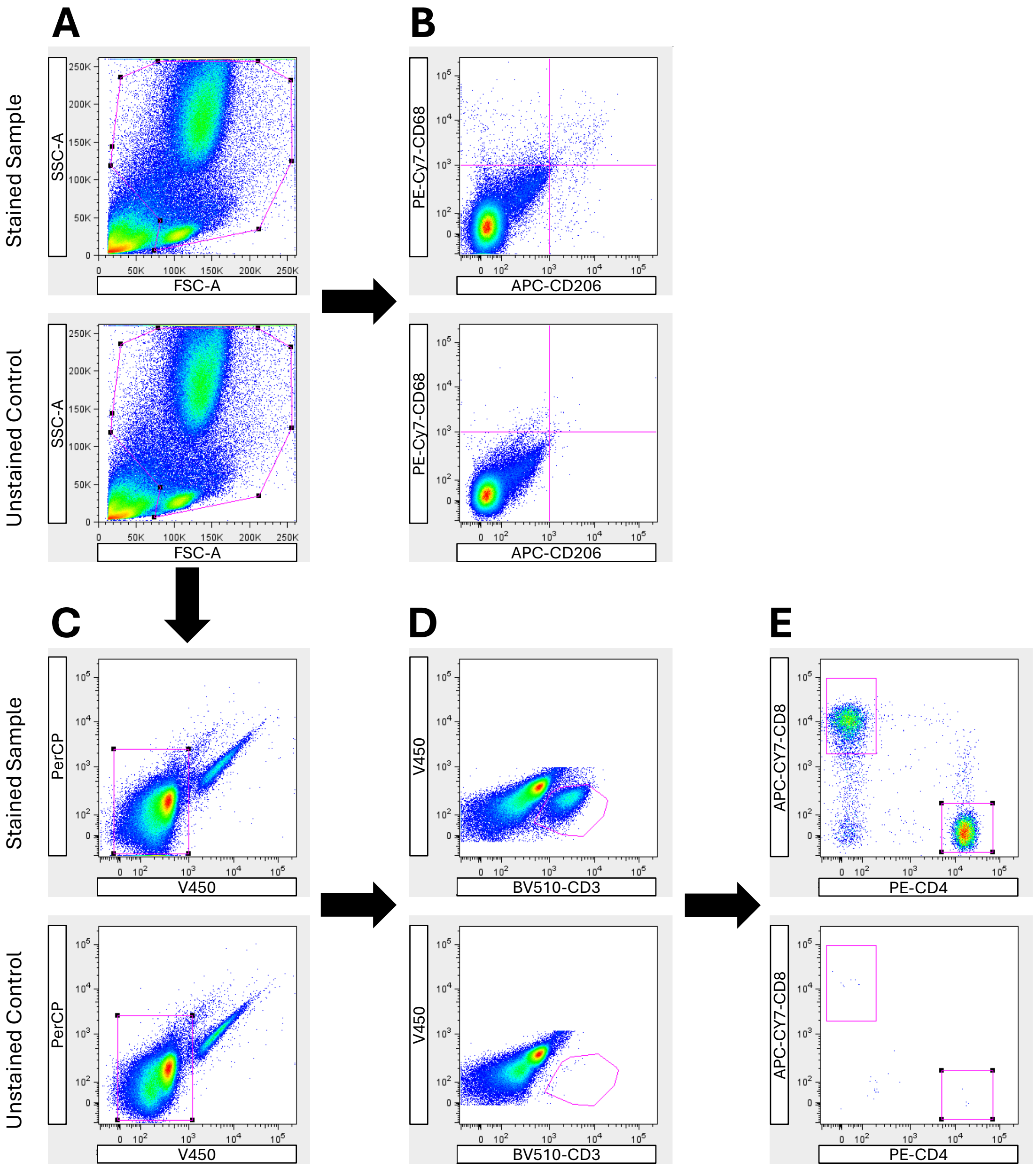


**Figure S1. Representative gating strategy for the identification of immune cell populations**

(**A**) Live cells were gated, and (**B**) macrophage ($CD68^{+}$) populations were identified as $CD206^{+}$ or $CD206^{-}$. (**C**) Unwanted events were eliminated by gating out the negative cells on the two dump channels, and (**D**) T cells were identified as $CD3^{+}$. (**E**) From the $CD3^{+}$ gate, T-cell subpopulations were identified as $CD8^{+}$ or $CD4^{+}$.

Adapted from Murphy et al. (2020), *Obesity*, 28(12):2310–2314, with permission from Wiley.

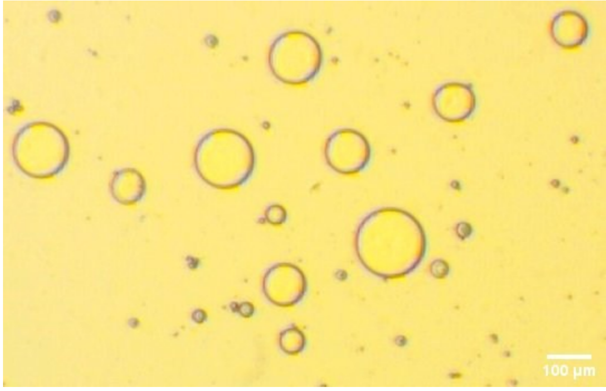


**Figure S2. Example phase-contrast microscopy image of adipocytes isolated by collagenase digestion**
